# Thermodynamic Analysis of Entropy Generation Minimization in Thermally Dissipating Flow Over a Thin Needle Moving in a Parallel Free Stream of Two Newtonian Fluids

**DOI:** 10.3390/e21010074

**Published:** 2019-01-16

**Authors:** Ilyas Khan, Waqar A. Khan, Muhammad Qasim, Idrees Afridi, Sayer O. Alharbi

**Affiliations:** 1 Faculty of Mathematics and Statistics, Ton Duc Thang University, Ho Chi Minh City 700,000, Vietnam; 2 Department of Mechanical Engineering, College of Engineering, Prince Mohammad Bin Fahd University, Al Khobar 31952, Saudi Arabia; 3 Department of Mathematics, COMSATS University Islamabad (CUI), Park Road, Tarlai Kalan, Islamabad-455000, Pakistan; 4 Department of Mathematics, College of Science Al-Zulfi, Majmaah University, Al-Majmaah 11952, Saudi Arabia

**Keywords:** entropy generation, Bejan number, thin needle, two Newtonian fluids, self-similar equations

## Abstract

This article is devoted to study sustainability of entropy generation in an incompressible thermal flow of Newtonian fluids over a thin needle that is moving in a parallel stream. Two types of Newtonian fluids (water and air) are considered in this work. The energy dissipation term is included in the energy equation. Here, it is presumed that *u*_∞_ (the free stream velocity) is in the positive axial direction (*x*-*axis*) and the motion of the thin needle is in the opposite or similar direction as the free stream velocity. The reduced self-similar governing equations are solved numerically with the aid of the shooting technique with the fourth-order-Runge-Kutta method. Using similarity transformations, it is possible to obtain the expression for dimensionless form of the volumetric entropy generation rate and the Bejan number. The effects of Prandtl number, Eckert number and dimensionless temperature parameter are discussed graphically in details for water and air taken as Newtonian fluids.

## 1. Introduction

The first law of thermodynamics is essentially the expression of the law of conservation of energy, i.e., during any interaction between a system and its surroundings, energy is always conserved. In other words, the first law of thermodynamics is concerned with the amount of energy and the conversion quantity of energy and its conversion from one form to another without stipulating the quality. In the design as well as development of different engineering products, quantity and specifying the quality of energy are considered key parameters.

The second law of thermodynamics offers us the needed tools to define not only the quality, but also the degree of degradation of energy throughout a process. Entropy or irreversibility is the key tool that helps measure energy quality. As per the second law of thermodynamics, when energy is being converted into something useful, energy losses always occur, thereby reducing the performance of devices used to convert energy. This means that energy is degraded (or destroyed) and is directly proportional to the generation of entropy. Therefore, there is always a decrease in the amount of available energy when entropy generation occurs in a system. Thus, how well a thermal system performs may be enhanced through the reduction of entropy generation. For this reason, it is critical to know how entropy generation is distributed during the process of thermodynamics so as to reduce its production. The performance and efficiency of most thermal systems is usually degraded because of the manifestation of irreversibilities and hence the entropy generation (${\dot{S}}_{gen}^{m}$), which is defined as the measure of the magnitude of the irreversibilities present during that process. The higher the degree of irreversibilities, the higher the rate of entropy generation. In other words, ${\dot{S}}_{gen}^{m}$ may be utilized in the establishment of the criteria needed to determine the performance of various engineering or thermal devices. The second law of thermodynamics has been used in various flow problems as well as thermal systems so as to help minimize the rate of entropy generation.

Bejan [1,2] was the first to examine the sources of entropy generation in a conventional heat transfer problem. The author established that the temperature gradient because of finite differences in velocity gradient and temperature was responsible for the production of entropy during the fluid flow process. Afterwards, several researchers followed the work of Bejan thereby studying how to apply the analysis of the second law thermodynamics in heat transfer and fluid flow problems so as to abate the generation of entropy [3,4,5,6,7,8,9,10,11,12,13,14,15,16,17,18]. Nonetheless, further exploration for such problems or aspects need to be conducted. This needle is a body shaped like a paraboloid of revolution with the axis in the direction of the incident flow. The diameter of thin needle is of the same order as that of velocity or thermal boundary layers developed. This particular shape of the boundary permits a similarity solution to exits and therefore enabled us to investigate the problem in detail. We consider in this problem a thin needle whose thickness is comparable to that of the boundary or thermal layers or smaller.

In 1967, Lee [19], studied the boundary layer flow over a thin needle. Such flows has many applications in aerospace and marine engineering, for example, flow of torpedoes, submarines, surface vehicles such as airplanes and many others. Chen and Smith [20] analyzed the forced convection heat transfers from non-isothermal thin needle. Ishaq et al. [21], studied numerically the boundary layer flow through a continuously moving thin needle that is in a parallel free motion. Grosan and Pop [22], investigated forced convection boundary layer flow past non-isothermal thin needles in nanofluids. They solved the resulting problem numerically for two types of nanoparticles, which were either nonmetallic or metallic, such as alumina (Al_2_O_3_) and copper (Cu) and water was taken as base fluid. Soid et al. [23] examined the analysis of boundary layer flow in a thin needle in continuous motion in a nanofluid. All the above thin needle problems focused on the heat transfer analysis only. However, there has been no attention given to the understanding of how entropy is generated with a thin needle in a parallel motion.

Therefore, the current study intends to carry out such an analysis. Appropriate transformation will be applied to help convert basic equations to self-similarity equations. Additionally, the shooting technique alongside the Runge-Kutta method will be applied to help obtain numerical solutions. To calculate the entropy generation number, we use the calculated temperature, velocity profile, and their gradients. Graphs will be used to show the impact of the various physical parameters on temperature distribution, velocity, entropy generation and the Bejan number.

## 2. Flow Analysis

**Figure 1:**
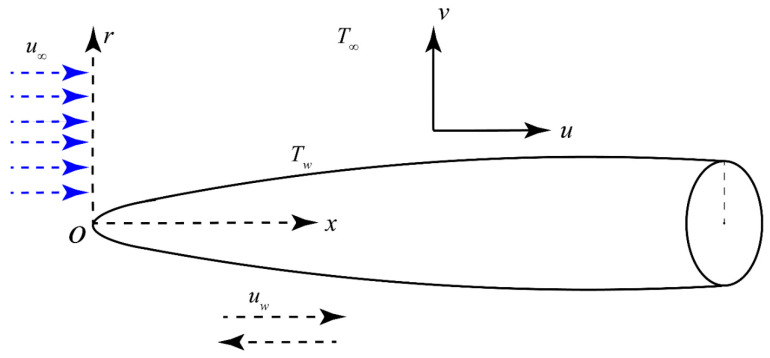
Flow model and coordinate system.

Consider the laminar steady flow of a dissipative fluid over a thin moving needle in a free stream. The thin needle is supposed to be moving with a constant velocity *u_w_* and the fluid in a stress-free region is moving with velocity *u*_∞_ as shown in Figure 1. Under the stated assumptions, the governing flow equations along with the boundary conditions take the following form [21,22,23,24]:

(1) $$\frac{\partial }{\partial x}\left(ru\right)+\frac{\partial }{\partial r}\left(rv\right)=0$$

(2) $$u\frac{\partial u}{\partial x}+v\frac{\partial u}{\partial r}=\frac{\nu }{r}\frac{\partial }{\partial r}\left(r\frac{\partial u}{\partial r}\right)$$

(3) $$\begin{array}{cc} u=u_{w},\;v=0, & \mathrm{at}r=R(x)\\ u\to u_{\infty } & \mathrm{as}r\to \infty \end{array}\}$$

where *r* and *x* show radial and axial coordinates respectively, *v* represents kinematic viscosity and 〈u,v〉, respectively, represent the components of velocity in the *x* and *r* directions.

The similarity variables are defined as [23]:

(4) $$\psi =\nu \;x\;f(\eta ),\eta =\frac{Ur^{2}}{\nu x}$$

where the stream function ψ is defined as $u=\frac{1}{r}\frac{\partial \psi }{\partial r}$ and $v=-\frac{1}{r}\frac{\partial \psi }{\partial x}.$ The velocity components *u* and *ν* identically satisfied the continuity equation. The composite velocity is defined as *U* = *u_w_* + *u*_∞_ ≠ 0 and the dimensionless stream function is denoted by *f*(*η*). By taking *η* = *a* (which represents the wall of the needle) in Equation (4), we get $R(x)={\left(\frac{a\nu x}{U}\right)}^{1/2}$ which describes the size and the shape of the surface of revolution).

Utilizing (2) and (3) we get the following form:

(5) $$ff^{\prime \prime }+2\left(f^{\prime \prime }+\eta f^{\prime \prime \prime }\right)=0$$

(6) $${\dot{S}}_{gen}^{\prime \prime \prime }=\frac{k}{T_{\infty }^{2}}\left[{\left(\frac{\partial T}{\partial r}\right)}^{2}+{\left(\frac{\partial T}{\partial x}\right)}^{2}\right]+\frac{\mu }{T_{\infty }}\left\{2\left[{\left(\frac{\partial v}{\partial r}\right)}^{2}+{\left(\frac{\partial u}{\partial x}\right)}^{2}\right]+{\left(\frac{\partial u}{\partial r}+\frac{\partial v}{\partial x}\right)}^{2}\right\}.\;f(a)=\frac{a\varepsilon }{2},\;f^{\prime }(a)=\frac{\varepsilon }{2},\;f^{\prime }(\infty )\to \frac{1-\varepsilon }{2}$$

where $\varepsilon =\frac{u_{w}}{u_{w}+u_{\infty }}$ represents the velocity ratio parameter. The local skin friction coefficient takes the following form:

(7) Cf(Rex)1/2=8a1/2f″(a)

where Rex=Uxν denotes local Reynolds number.

### 2.1. First Law Analysis

The energy equation in cylindrical coordinates for dissipative fluid flow over a thin needle is:

(8) $$u\frac{\partial T}{\partial x}+v\frac{\partial T}{\partial r}=\frac{\alpha }{r}\frac{\partial }{\partial r}\left(r\frac{\partial T}{\partial r}\right)+\frac{\nu }{c_{p}}{\left(\frac{\partial u}{\partial r}\right)}^{2}$$

where *T*, *α*, *ν* and *c_p_* represent Equation (4), Equation temperature distribution, thermal diffusivity, kinematic viscosity and specific heat at constant pressure. The imposed boundary conditions are:

(9) $$\begin{array}{l} T=T_{w}\;\mathrm{at}\;r=R(x)\\ T\to T_{\infty }\;\mathrm{as}\;r\to \infty \end{array}\}$$

Further, it is supposed that *T_w_* > *T*_∞_ (heated needle) and the dimensionless temperature *θ*(*η*) is given by:

(10) $$\theta \left(\eta \right)=\frac{T-T_{\infty }}{T_{w}-T_{\infty }}$$

Using Equations (4) and (10), Equations (8) and (9) reduce to:

(11) $$\eta \theta^{\prime \prime }+\theta^{\prime }\left(1+0.5\mathrm{Pr}f\right)+4\eta Ec\mathrm{Pr}{f^{\prime \prime }}^{2}=0$$

(12) θ(a)=1,θ(η→∞)=0

where Pr=να and Ec=U2cp(Tw−T∞) are the Prandtl and Eckert numbers, respectively. The expression for the local Nusselt number *N_u_* = (Re*_x_*)^−1/2^ is:

(13) Nu(Rex)−1/2=−2a1/2θ′(a)

### 2.2. Second Law Analysis

The complete expression of entropy generation for two-dimensional flow with thermal dissipation in cylindrical coordinate is:

(14) $${\dot{S}}_{gen}^{\prime \prime \prime }=\frac{k}{T_{\infty }^{2}}\left[{\left(\frac{\partial T}{\partial r}\right)}^{2}+{\left(\frac{\partial T}{\partial x}\right)}^{2}\right]+\frac{\mu }{T_{\infty }}\left\{2\left[{\left(\frac{\partial v}{\partial r}\right)}^{2}+{\left(\frac{\partial u}{\partial x}\right)}^{2}\right]+{\left(\frac{\partial u}{\partial r}+\frac{\partial v}{\partial x}\right)}^{2}\right\}$$

Utilizing boundary layer approximations Equation (14) reduces to:

(15) $${\dot{S}}_{gen}^{\prime \prime \prime }=\frac{k}{T_{\infty }^{2}}{\left(\frac{\partial T}{\partial r}\right)}^{2}+\frac{\mu }{T_{\infty }}{\left(\frac{\partial u}{\partial r}\right)}^{2}.$$

The terms $\frac{k}{T_{\infty }^{2}}{\left(\frac{\partial T}{\partial r}\right)}^{2}$ and $\frac{\mu }{T_{\infty }}{\left(\frac{\partial u}{\partial r}\right)}^{2}$ respectively represent the entropy production by virtue of heat transfer ${\dot{S}}_{prod,\Delta T}^{\prime \prime \prime }$ and fluid friction.

Using Equations (4) and (10), Equation (14) becomes:

(16) Ns=S˙prod‴kΩ2TηRex/x2=θ′2+4Brf″2=NΔT+NFric

where *Br* = EcPr/Ω*_T_* (modified Brinkman number), *N*_Δ*T*_ = *θ*^’2^ (conductive irreviersibility), *N_Fric_* = 4*Brf*″^2^, (S˙prodm)c=4kΩT2ηRexx2(characteristic entropy).

Bejan [1] introduced the expression of irreversibility distribution ratio as given below:

(17) $$\Phi ={\dot{S}}_{prod,Fric}^{\prime \prime \prime }/{\dot{S}}_{prod,\Delta T}^{\prime \prime \prime }$$

It is noteworthy to underline that when Φ>1, the fluid friction irreversibility *Ns_f_* is the major factor whereas when 0<Φ<1 the heat transfer irreversibility *Ns_h_* is the dominant one. When Ф = 1, the improvement secondary to heat transfer (*Ns_h_*) and to fluid friction (*Ns_f_*) are equal.

Equation (18) takes the form:

(18) $$\Phi =\frac{4Br{f^{\prime \prime }}^{2}}{{\theta^{\prime }}^{2}}$$

The irreversibility ratio also known as Bejan number is given by:

(19) $$Be=\frac{{\dot{S}}_{prod,\Delta T}^{\prime \prime \prime }}{{\dot{S}}_{prod}^{\prime \prime \prime }}=\frac{\frac{k}{T_{\infty }{}^{2}}{\left(\frac{\partial T}{\partial r}\right)}^{2}}{\frac{k}{T_{\infty }{}^{2}}{\left(\frac{\partial T}{\partial r}\right)}^{2}+\frac{\mu }{T_{\infty }}{\left(\frac{\partial u}{\partial r}\right)}^{2}}$$

Using similarity transformations, Equation (20) takes the following form:

(20) $$Be=\frac{{\theta^{\prime }}^{2}}{{\theta^{\prime }}^{2}+4Br\text{​}{f^{\prime \prime }}^{2}}$$

## 3. Results and Discussions

**Figure 2:**
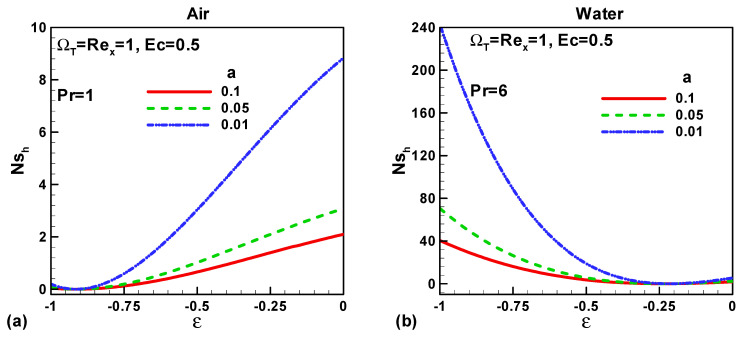
Variation of *Ns_h_* when *u*_∞_ > 0 and the *ε* = 0 for (**a**) air and (**b**) water.

**Figure 3:**
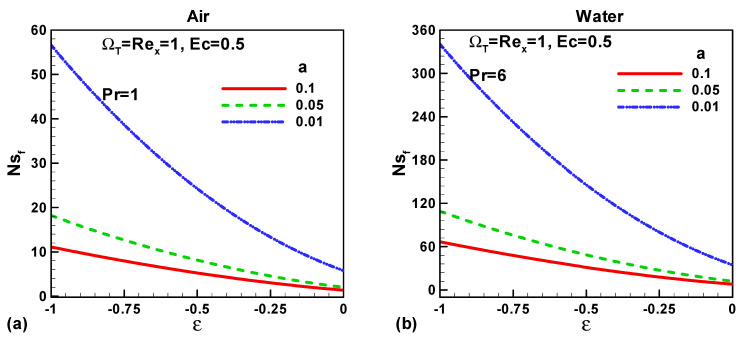
Variation of *Ns_f_* when *u*_∞_ > 0 and the *ε* < 0 for (**a**) air and (**b**) water.

**Figure 4:**
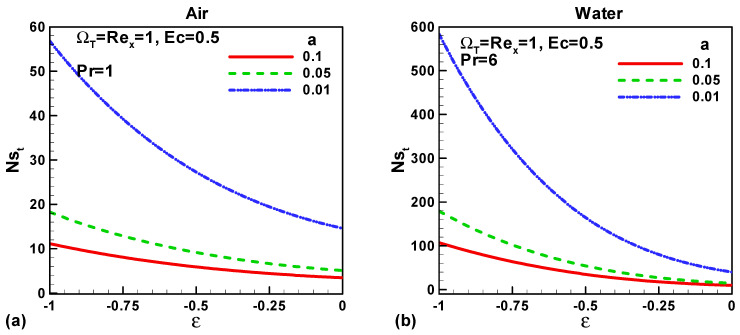
Variation of *Ns_t_* when *u*_∞_ > 0 and the *ε* < 0 for (**a**) air and (**b**) water.

**Figure 5:**
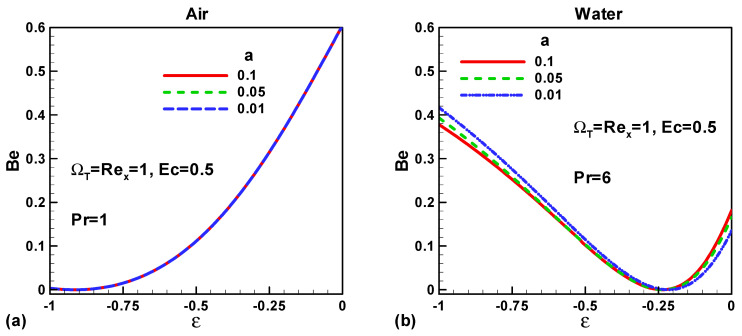
Variation of *Be* when *u*_∞_ > 0 and the *ε* < 0 for (**a**) air and (**b**) water.

**Figure 6:**
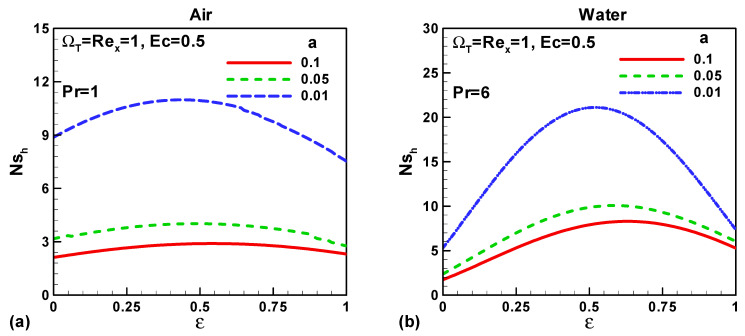
Variation of *Ns_h_* when (0 < *ε* < 1) for (**a**) air and (**b**) water.

**Figure 7:**
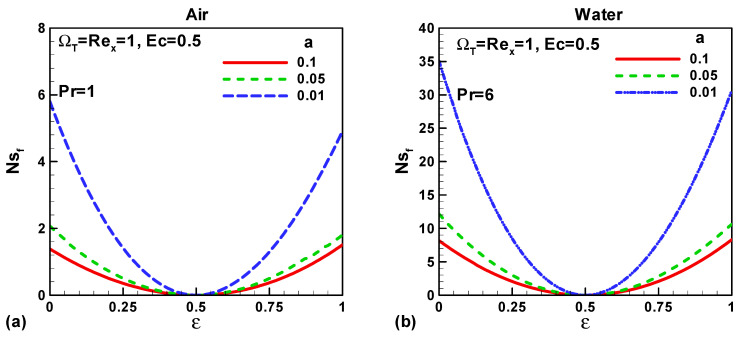
Variation of *Ns_f_* when (0 < *ε* < 1) for (**a**) air and (**b**) water.

**Figure 8:**
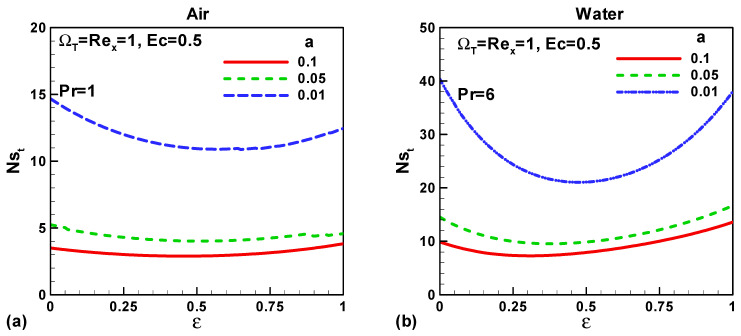
Variation of *Ns_t_* when (0 < *ε* < 1) for (**a**) air and (**b**) water.

**Figure 9:**
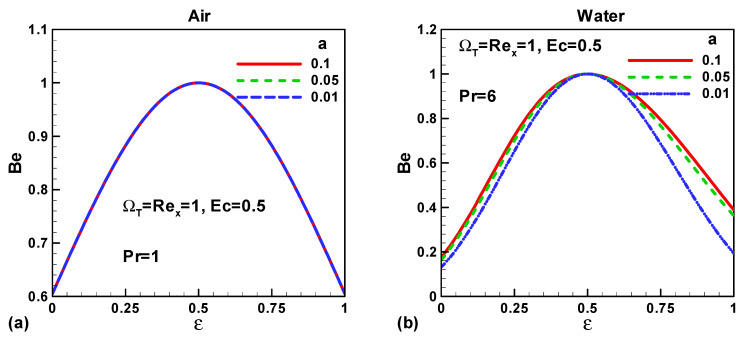
Variation of *Be* when (0 < *ε* < 1) for (**a**) air and (**b**) water.

In this section, our key concern is to look at the impacts of the chief physical parameters on the quantities of interest. Therefore Figure 2, Figure 3, Figure 4, Figure 5, Figure 6, Figure 7, Figure 8 and Figure 9 are given. Figure 2a,b show the variation of entropy generation as a result of heat transfer and viscous irreversibility with dimensionless size of thin needle (*a*) and velocity ratio parameter *ε* < 0 for both type of fluids (air and water), respectively. It is found that conduction irreversibility increases for both air and water as fluid with decreasing size of the needle. For a fixed thin needle size, the conduction irreversibility increases for air and decreases for water with decreasing (in an absolute sense) velocity ratio parameter. Furthermore, the comparison also reveals that the entropy generated due to heat transfer in water is more than in air and this is because of the high Prandtl number. Similar effects are observed for fluid friction irreversibility as depicted in Figure 3a,b. The total entropy generation (*Ns*)*_t_* increases with the diminishing size of the thin needle as depicted in Figure 4a,b and this is because of the increasing velocity and temperature gradients. Figure 4a,b also reveal that the total entropy generated in water is more than in air. Furthermore, for a fixed value of “*a*” the entropy decreases as the needle velocity decreases or the free stream velocity increases, so by increasing the free stream velocity or by decreasing the velocity of the thin needle one can minimize the entropy generation, which is the main goal of the second law analysis, the minimization of entropy generation.

Figure 5a,b describe the variation of Bejan number (*Be*) with needle size “*a*” and velocity ratio parameter *ε* < 0 for air and water, respectively. It is found that the Bejan number is independent of “*a*” if the working fluid is air. In the case of water, the Bejan number increases with decreasing size of the needle and decreases with the falling values of the velocity ratio parameter (up to *ε* ≈ −0.25). The Bejan number is less than 0.5 in the case of water therefore the contribution of fluid friction is dominant over the contribution of heat transfer as shown in Figure 5b.

For both types of fluid, air and water, the entropy generated because of heat transfer increases with decreasing magnitude of “*a*”. Physically, with decreasing needle size the thermal boundary layer thickness reduces, i.e., the temperature gradient increases which in turn enhances the heat transfer irreversibility. For fixed vale of “*a*” entropy due to heat transfer attains its peak value and then decreases as the velocity ratio parameter increase. This behaviour is examined for both type of fluid air and water as portrayed in Figure 6a,b. For any fixed value of *ε* except *ε* = 0.5 the contribution of fluid friction in entropy generation increases with decreasing dimensionless size of the needle. At *ε* = 0.5 the fluid friction plays no role in the entropy generation. Physically, when *ε* = 0.5 the free stream velocity is equal to the needle velocity (fluid and needle are relatively at rest) and therefore the velocity gradients cancel out. The total entropy generation increases with the decreasing size of needle for both type of fluids, air and water, as shown in Figure 8a,b. These figures also describe that more entropy is generated in water as compared to air. Furthermore, for any fixed size of the thin needle (let’s take *a* = 0.1) the entropy decreases up to the fixed value of velocity ratio parameter *ε* = 0.5 and then increases. It is also found that as the size of the thin needle decreases the total entropy generated in Sakiadis flow dominating the entropy generated in Blasius flow.

Figure 9a,b show the effects of needle size and velocity ration parameter (0 ≤ *ε* ≤ 1) on Bejan number for both type of fluids, air and water, respectively. The Bejan number remains constant with the variation of needle size for air and decreases for water. Furthermore, as the velocity ratio parameter increases the Bejan number attains its maximum value and then decreases. At *ε* = 0.5, heat transfer irreversibility is completely dominant to viscous irreversibility irrespective of the size of the thin needle and fluid type. This is because, at *ε* = 0.5 the velocity gradients vanish and therefore viscous effects have no contribution to entropy generation.

## 4. Closing Remarks

The effects of viscous dissipation on entropy generation in a fluid flow over a thin needle is studied. The following are the key conclusions of the present study:

- Heat transfer and fluid friction irreversibility increases with the decreasing size of the thin needle for both type of fluids air and water.
- Total entropy enhances with the reduced size needle.
- Entropy generated due to heat transfer and fluid friction in water is more than in air.
- When *ε* < 0, entropy can be minimized either by increasing the free stream velocity or by decreasing the needle velocity.

## Nomenclature

a	Constant
Be	Bejan Number [−]
Br	Brinkman number [−]
$c_{p}$	Specific heat $\left[{\mathrm{JKg}}^{-1}{\;K}^{-1}\right]$
Ec	Eckert number [−]
f	Dimensionless stream function
k	Thermal conductivity $\left[{\mathrm{Wm}}^{-1}{\;K}^{-1}\right]$
Pr	Prandtl number [−]
R(x)	Shape and size of the needle [m]
$Re_{x}$	Local Reynold number [−]
T	Temperature field [K]
U	Composite velocity [${m\;s}^{-1}$]
$u_{w},u_{\infty }$	Velocity of needle and free stream, respectively [${m\;s}^{-1}$]
u,v	Velocity components in axial and radial directions, respectively [m]
x,r	Spatial coordinates measured in axial and radial directions, respectively [m]
θ	Non-dimensional temperature [−]
ρ	Density of fluid $\left[{\mathrm{kg}\;m}^{-3}\right]$
μ	Dynamic viscosity $\left[{\mathrm{kg}\;m}^{-1}{\;s}^{-1}\right]$
Ψ	Dimensional stream function $\left[m^{2}\;s^{-1}\right]$
ε	Velocity ratio parameter
